# Targeting and Understanding HIV Latency: The CRISPR System against the Provirus

**DOI:** 10.3390/pathogens10101257

**Published:** 2021-09-28

**Authors:** Gloria Magro, Arianna Calistri, Cristina Parolin

**Affiliations:** Department of Molecular Medicine, Microbiology and Virology Unit, University of Padua, 35121 Padua, Italy; gloria.magro@phd.unipd.it

**Keywords:** HIV latency, CRISPR-Cas, gene editing, CRISPR screens, host dependency factors

## Abstract

The presence of latently infected cells and reservoirs in HIV-1 infected patients constitutes a significant obstacle to achieve a definitive cure. Despite the efforts dedicated to solve these issues, the mechanisms underlying viral latency are still under study. Thus, on the one hand, new strategies are needed to elucidate which factors are involved in latency establishment and maintenance. On the other hand, innovative therapeutic approaches aimed at eradicating HIV infection are explored. In this context, advances of the versatile CRISPR-Cas gene editing technology are extremely promising, by providing, among other advantages, the possibility to target the HIV-1 genome once integrated into cellular DNA (provirus) and/or host-specific genes involved in virus infection/latency. This system, up to now, has been employed with success in numerous in vitro and in vivo studies, highlighting its increasing significance in the field. In this review, we focus on the progresses made in the use of different CRISPR-Cas strategies to target the HIV-1 provirus, and we then discuss recent advancements in the use of CRISPR screens to elucidate the role of host-specific factors in viral latency.

## 1. Introduction

The human immunodeficiency virus (HIV)—after almost 40 years since its discovery and after many economic [1] and scientific efforts made to put an end to its epidemic—still constitutes a major public health issue. At the end of 2020, there were nearly 38 million people worldwide living with the virus (WHO). Fortunately, nowadays, people with HIV can lead ‘normal’ lives, thanks to the development of combined antiretroviral therapy (cART) as the standard of care [2]. cART consists of lifelong administration of a cocktail of drugs (generally two or three) to suppress viral replication to undetectable levels and to reduce the risk of viral transmission [3]. However, this therapy does not represent a definitive cure and the infection can persist, albeit in a chronic state, throughout a person’s lifespan. 

What makes a definitive cure for—or an effective vaccine against—HIV difficult to achieve is the particular lifecycle of the virus and its unique interactions with the target cells. HIV is an enveloped lentivirus whose genome consists of two identical single-stranded RNAs, encoding for the main viral proteins Gag (structural proteins, such as the capsid-forming p24 protein), Pol (viral enzymes including reverse transcriptase, integrase, and protease) and envelope glycoproteins (Env), polyproteins processed by proteolytic cleavage. In addition, accessory regulatory proteins are produced, such as trans-activator of transcription (Tat), promoting transcriptional elongation of the full-length viral RNA, and Rev, involved in the transport of unspliced/partially spliced viral RNAs from the cell nucleus to the cytoplasm. The virus enters the target cells by binding the cell surface CD4 receptor and a co-receptor, either CCR5 (especially relevant in the first phase of the infection) or CXCR4. Next, viral RNA is released in the cell, retrotranscribed to DNA by the viral reverse transcriptase, and transported to the cell nucleus. The viral DNA presents at its ends two identical long terminal repeats (LTRs), which work both as a promoter and a polyadenylation signal, and are indispensable for the binding of the viral integrase and the subsequent integration of the viral DNA (hence, known as “provirus”) in the host cell genome [4]. Once integrated, the provirus exploits the cell machinery for its replication.

HIV targets mainly the CD4^+^ cells of the immune system (among which T-cells and macrophages). In most of the T-cells, viral infection is productive, causing cell death followed by a decrease in the CD4^+^ cell count over time, until the onset of the immunodeficiency syndrome [5]. On the other hand, long-lived memory T-cells are able to enter a quiescent state in which the transcriptional state is poised, due, for example, to epigenetic alterations [6]. In this context, key transcription factors for HIV replication are not available and the provirus enters in a non-replicative state, known as latency (for a comprehensive review on HIV latency see [7,8,9]). Thus, HIV can evade clearance by the immune system and the effect of the antiretroviral therapies, which mainly function on the actively replicating virus. If therapy is suspended, and the quiescent cells’ milieu becomes permissive for replication, the virus can rebound [10].

**Figure 1:**
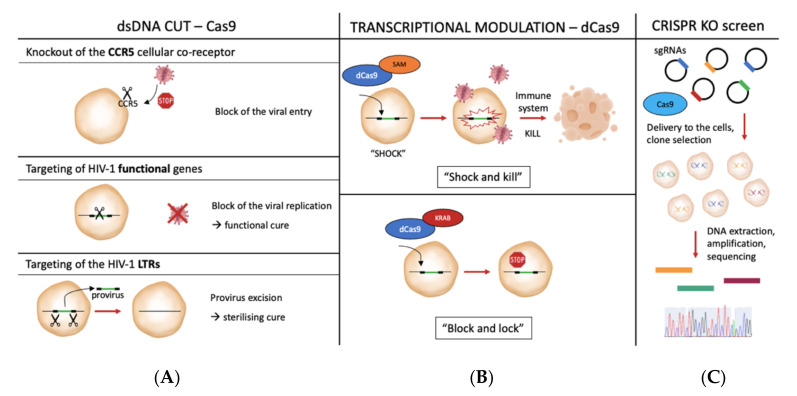
Novel CRISPR/Cas approaches to target HIV-1 or host-specific genes involved in the virus lifecycle. (**A**), some approaches based on the double-strand DNA (dsDNA) endonuclease activity of the Cas9, to target either the CCR5 co-receptor, thus blocking viral entry, or functional genes in the HIV-1 provirus (therefore, impairing viral replication) or the long terminal repeat (LTR) sequences to promote the provirus excision. (**B**), potential strategies to modulate provirus transcription by using the dCas9 protein combined with either transcriptional activators or transcriptional repressors (“shock and kill” or “block and lock” strategies, respectively), further explained in Section 4 of the present review. (**C**), a schematic representation of the general steps of a CRISPR knockout screen, which may be employed to identify novel host genes involved in the viral replication cycle. SAM stands for synergistic activator mediator, while KRAB stands for Krüppel-associated Box.

In the last few years, the development of gene editing technologies, and especially of the most recent and versatile clustered regularly interspaced short palindromic repeats-CRISPR associated protein (CRISPR-Cas) system, opened up new possibilities in the field of HIV research, with the advancements of novel approaches to (i) directly target the virus in its integrated or non-integrated form; (ii) modulate its expression (e.g., using the dCas9 in “block and lock” [11] or “shock and kill” [12] strategies); (iii) target host genes involved in the viral entry and replication cycle (such as the CCR5 co-receptor [13]); (iv) discover new host-specific molecular targets or new host genes involved in the viral lifecycle (e.g., CRISPR screening [14]) (Figure 1). 

Some of these strategies showed promising results, in both in vitro and in vivo studies, and are moving towards the clinical stages [15]. To have an idea about the impact that the CRISPR technique has in this field—it is exemplary that the first ever (and unanimously criticized) use of the gene editing technology to edit human embryos, generating “gene-edited babies”, was applied to perform a knockout of the CCR5 co-receptor [16]. This approach was aimed at conferring to the newborns resistance to HIV infection. However, the lack of a real consideration of the possible negative impact of this knockout on the babies’ lives led to ethical debates on how to discriminate what we could potentially do, from what we should effectively do with this potent tool [17].

Aside from this controversial application, currently, many studies are investigating the possibility of targeting the CCR5 co-receptor to develop a functional cure against HIV [18]. A functional cure is defined as the suppression of the virus replication. Viral load remains undetectable in the absence of cART without the need of completely eliminating the virus from the organism (the complete elimination would be defined as a sterilizing cure). The idea that this could be sufficient at preventing the progression to AIDS as well as viral spread to other individuals, came from the observations of the Berlin [19] and London [20] patients who, after bone marrow transplantation from donors homozygous for the Δ32 deletion in the CCR5 co-receptor, achieved remission. Based on these findings, gene editing technologies, such as zinc-finger nucleases (ZFN) [21], transcription activator-like effector nucleases (TALEN) [22], and more recently, CRISPR-Cas [23], have been developed to edit ex vivo the CCR5 gene, and make the cells resistant to infection, an approach that has also been tested in clinical trials, giving promising preliminary results regarding the safety profile. However, the latter strategy cannot eliminate the virus from the infected cells; hence, this is why different investigations have exploited the possibility to directly target the actively replicating virus or the latent provirus. 

Given the numerous possible applications, in this review, we focus on how the CRISPR-Cas system was adopted in the last few years in the development of novel strategies to eradicate HIV infection. In particular, we focus on the approaches exploited to modulate provirus replication or to excise it from the host cell genome. Furthermore, we discuss the CRISPR-Cas system application to the study of the complex viral/cellular interplay, which allows the establishment/maintenance of viral latency, which is still one of the less understood aspects of viral pathogenesis. 

## 2. A Brief Overview of the CRISPR-Cas System

### 2.1. The CRISPR-Cas System

**Figure 2:**
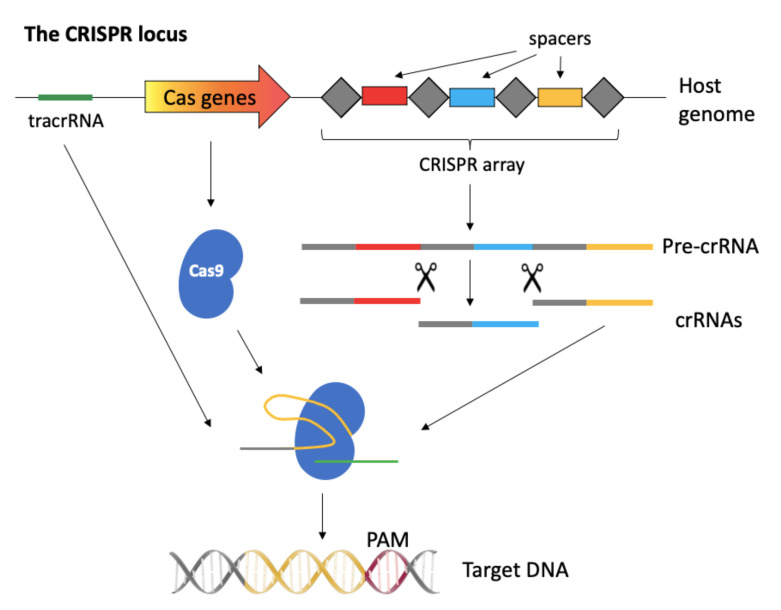
Schematic representation of the CRISPR-Cas system in bacteria. PAM stands for protospacer adjacent motif.

The CRISPR-Cas system derives from bacteria, where it works as an adaptive defense mechanism against infections. Within the bacterial genome, in fact, the CRISPR-Cas locus is formed by a series of repeated sequences interspaced with short unique sequences (spacers) derived from bacteriophages or plasmids encountered in past infections, forming the CRISPR array. When the cells uptakes a foreign DNA previously encountered, the CRISPR array is transcribed and processed in mature crRNAs, which complex with the Cas nuclease along with a trans-activating crRNA (tracrRNA), and bind to the complementary invader DNA, cutting it, and protecting the cell from the infection. For the cut to be specific against the foreign DNA and not to be directed to the bacterial CRISPR array, the DNA-binding activity of the complex is restricted to those sequences that are flanked by a protospacer adjacent motif (PAM), a very short sequence present in the invader DNA, but not in the CRISPR array (Figure 2).

Given its ability to perform a double-strand cut on DNA sequences with a sequence specificity dependent on the short crRNA, CRISPR-Cas was soon adapted as a gene editing system in mammalian cells, by fusing the crRNA together with the tracrRNA to form a single guide RNA (gRNA or sgRNA). gRNAs can virtually recognize any 20 base pair sequence and lead to its cut, provided that the target is flanked by a PAM sequence, such as “NGG” (where N stands for any base) in the case of SpCas9 (*Streptococcus pyogenes* Cas9), the firstly and most commonly employed nuclease [24] (for a more in-depth review on the CRISPR-Cas9 system see [25]). Following the double-strand cut, cellular DNA can be repaired through a non-homologous end-joining repair pathway (NHEJ), characterized by error-prone joining of the cleaved DNA. This mechanism leads to indels and frameshifts and, thus, to the possible knockout of the targeted gene. Otherwise, the DNA repair can occur through the less efficient homology-directed repair (HDR), characterized by the use of a homology template for a precise repair and therefore for the possible knock-in of a desired DNA sequence [26]. As it will be presented in this review, most of the studies employing CRISPR-Cas against HIV have exploited the most efficient and most commonly pathway used by the cells, the NHEJ, to introduce indels and, thus, to disrupt the coding sequences of viral/host genes.

To perform a double-strand DNA cut, Cas9 is provided by two nuclease domains, RuvC-like and HNH, each cleaving one strand of the target DNA sequence. Deletion of one of these domains, transforms Cas9 into a nickase able to perform a single strand cut. The deletion of both domains generates the so-called dCas9 (or catalytically “dead” Cas9), which loses its nuclease function but retains its ability to bind to specific DNA sequences, guided by the gRNA. The dCas9 can be coupled with transcription modulators, representing a convenient system to perform epigenetic modifications or transcriptional regulation [27], as it will be further described in Section 4.

### 2.2. Delivery Systems

Cas9 and gRNA components of the CRISPR system can be introduced in target cells in different ways, depending on the approach adopted and if stable or transient expression of the Cas9 and/or the gRNAs are needed. The nuclease and the gRNA/s can be encoded by a single construct, by separate plasmids, or can be provided as RNAs. Alternatively, the Cas9 nuclease can be directly delivered along with the gRNA as a ribonucleoprotein complex. In this regard, various systems can be employed, including standard cell transfection techniques and physical methods, such as microinjection [28] or electroporation [29], with the advantage of having a limited in time permanence of the components within the cells. However, these systems are not suited for in vivo applications. On the other hand, non-viral delivery vehicles, such as nanoparticles/lipid-nanoparticles or viral vector-based systems, can be applied both in vitro and in vivo. Among viral vectors, lentiviral vectors (LVs) have the advantage that their size allows accommodating both the Cas9 nuclease and gRNA-coding sequences in the same vector. Furthermore, they can be designed as integrative or non-integrative, thus conferring a transient or stable expression of the CRISPR components in the cells. While the stable expression of the gRNAs and nuclease may be desirable for some applications, the downside is that it could lead to increased off-target effects, i.e., the cut in DNA sequences other than the gRNA-targeted ones [30]. Among non-integrative viral vectors, adeno-associated viral vectors (AAVs) are smaller than LVs, rendering the packaging of the Cas9 and gRNA in the same particle more challenging. Nevertheless, the presence of several serotypes to target different cell types and the transgene long-term expression in the absence of integration make these vectors very attractive for in vivo applications [26].

### 2.3. Cas Nuclease Variants

Other than the SpCas9, nuclease variants isolated from different bacteria can be used, conferring a great versatility to the system and facilitating the development of effective delivery strategies, a crucial step when targeting HIV provirus in vivo. For example, the SaCas9 (*Staphylococcus aureus*-derived) is more manageable for AAV vector packaging, being around 1 kilobase shorter than the first-employed SpCas9. The possibility to adopt AAV for the delivery is useful to target the integrated provirus in different tissues and reservoirs in vivo [31,32]. In addition to SaCas9 nuclease, Cas12a, formerly known as Cpf1, is another nuclease, which not only is smaller than SpCas9, but can also better accommodate the combination of multiple crRNAs under the transcriptional control of a single Pol III promoter. The performance of the latest nuclease in targeting different HIV-1 sequences was assessed by Gao and co-workers, showing a more sustained antiviral activity when compared to Cas9, at least in vitro [33]. Data showed that, in stably transduced cells, Cas12a efficiently inhibits HIV-1 replication over time, even when one single crRNA is adopted. This result may be ascribed to the specific Cas12a architecture, as well as to the distinct mutation profile induced by its activity. Indeed, while Cas9 activity results in blunt double-strand cuts, Cas12a leads to staggered cuts in the dsDNA. Furthermore, additional nucleases, such as the RNA-targeting Cas13, have been tested in the context of HIV-1 infected cells [34].

While many nucleases have been (and are being) developed, the choice of what to use will finally depend on the context of application.

### 2.4. Considerations on the Selection of the HIV-1 Targets

When designing a CRISPR-Cas9 gene editing strategy, it is of utmost importance to choose the right target sequence, or combination of targets, and to carefully design the gRNAs. This is even more true in the context of HIV-1, as from the earliest studies it became apparent that the success of the approach is strongly dependent on the outline of these elements.

In the first place, the choice of the target HIV-1 sequence relies upon the final objectives of the study, i.e., if the aim is to accomplish a functional or a sterilizing cure [35]. For instance, the targeting of HIV-1 regulatory genes (such as *tat*, *rev*) or of host-specific genes leads to the suppression of the viral replication and to the so-called functional cure. As mentioned in the introduction, in this case, viral replication is inhibited and the cells of the immune systems are protected, but the virus is not completely eradicated. For example, in a study by Ophinni and co-workers [36], gRNAs were designed against *tat* and *rev* sequences, leading to the efficient inhibition of HIV-1 replication, both in persistently and latently infected cell lines.

Another possibility is to target the viral LTRs, thus leading not only to the disruption of the viral replication, but eventually also to the excision of the entire provirus from the host cell, to achieve a sterilizing cure. This approach was tested in vivo [37] in a humanized bone marrow, liver, thymus (BLT) [38] mouse model of latent infection, by using AAV expressing SaCas9 and multiple LTR- and *gag*/*pol*-targeting gRNAs. Results demonstrated an efficient excision of the provirus in different tissues. Interestingly, in this study the authors also compared the excision efficiency of a duplex or a quadruplex all-in-one vector targeting LTR and regulatory sequences in neural stem cells isolated from HIV-1 infected Tg26 mice (a transgenic mouse model harboring in different tissues an integrated replication-deficient HIV genome [39]). Data indicated that the quadruplex approach was more efficient than the duplex one in inducing viral excision [37]. This study will be further discussed in Section 3.

### 2.5. Considerations on the Design of the gRNAs

**Figure 3:**
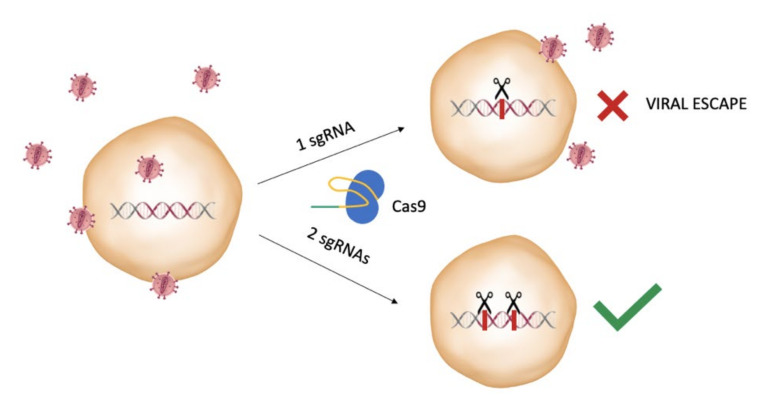
A combinatorial CRISPR/Cas9 approach is needed to effectively block the viral replication. While the use of a single HIV-1-targeting sgRNA may lead to the generation of resistant mutants, the use of a dual sgRNA approach is able to stop the viral replication, avoiding generation of escape mutants.

When selecting which proviral sequence to target, careful design of the gRNAs is important to avoid possible off-target effects and the potential emergence of resistant mutants. In fact, it was demonstrated by Wang and collaborators that targeting the HIV-1 provirus with a single gRNA could not only lead to resistance, but also to NHEJ repair mechanism at the cut site, facilitating viral escape [40]. This occurs since some of the indels introduced at the NHEJ site may lead to a still functional provirus harboring mutated sites that prevent the Cas9 and gRNA complex binding and cleavage, thus originating CRISPR-resistant mutants. Soon after, the same group demonstrated that this problem could be overcome by using a combinatorial approach, i.e., the targeting of more than one site in the proviral genome, as it was previously investigated employing the interfering RNA approaches. The efficiency of different gRNA combinations was assessed by targeting both the LTRs and functional genes and determining the ability of this strategy in delaying or interfering with viral breakthrough in infected T-cell cultures [41] (Figure 3).

Furthermore, the problem of the emergence of resistant viruses may be overcome by targeting highly conserved sequences, as it is less probable that a functional resistant mutant could be generated. It is well known that HIV-1 displays a great inter- and intra-patient variability, so bioinformatic pipelines have come in handy to predict what are the most conserved sequences among different variants and, thus, select the possible most efficient broad-spectrum gRNAs in order to be effective in as many patients as possible [42,43].

Many gRNAs or combination of gRNAs for HIV-1 gene editing approaches have been selected and tested up to this day in different in vitro and in vivo studies. However, to confirm their efficiency and on-target specificity for a possible therapeutic application, a validation of the system in a proper experimental setting and using the appropriate model is necessary. For example, it has been shown that some off-target sites, which are predicted in silico and may be observed in in vitro assays, such as the CIRCLE-seq, may lead to different results when using the system in vivo [44]. The availability of advanced validation tests should enable faithful interpretation of the data and facilitate moving to the clinic.

## 3. The CRISPR System for Editing of HIV Sequence in Latency Models

CRISPR-Cas9 can virtually target every step of the HIV lifecycle [45] due to its versatility. Furthermore, this system proved to be especially effective in targeting the provirus in latently infected cells. The first proof-of-principle that CRISPR-Cas9 could be exploited to treat HIV infection, at least in vitro, was reported in 2013 [46], just one year after the discovery of the CRISPR-Cas9 system as a programmable genome editing tool [24]. It was the first demonstration that targeting of the HIV-1 LTR by CRISPR-Cas9 could block LTR-driven gene expression and lead to the excision of the region encompassed between the LTRs, with potential eradication of the provirus. The authors also tested the system in human CD4^+^ lymphoblastoid Jurkat cells mimicking HIV-1 latency. Specifically, upon induction of viral replication by treatment with latency reversing factors, they also showed, in this experimental setting, a CRISPRCas9-mediated inhibition of viral gene expression [46]. Overall, these results proved that the system is successful against the replicating virus; however, there was no information on its ability to access transcriptionally silent sites. This aspect was investigated by Zhu and co-workers [47], who tested different LTR-, *pol*-, and *tat*/*rev*-targeting gRNAs in single or in combination in a different cellular model of HIV latent infection. The authors showed that the cell pre-treatment with transcriptional activators, such as TNFα, did not improve CRISPR-Cas9 antiviral effect with respect to the non-pre-treated cells. These results suggested the ability of the nuclease to act on the latent provirus. 

Based on these findings, Liao and collaborators [48] hypothesized and experimentally confirmed that the CRISPR-Cas9 system could be able to act not only against the integrated virus, active or latent, but also against the pre-integration form of the viral DNA, possibly constituting an effective intracellular defense against HIV-1. Importantly, a pivotal study moved from cell lines to primary cells by adopting primary CD4^+^ T-cells and PBMCs derived from both healthy and HIV-1-infected individuals [49]. In particular, in patient-derived cells, the treatment with a lentivirus-delivered CRISPR-Cas9 system led to a reduction up to 92% of the viral copy number, and up to 71% in p24 production. Moreover, potential off-target effects were extensively investigated, demonstrating that the system does not cause genotoxicity to the host DNA.

Although the in vitro studies have mainly focused on T-cells [50], HIV-1 reservoir cell compartment is heterogeneous and is not composed only by T-cells. Interestingly, suppression of the HIV-1 provirus by adopting an LTR-targeting Cas9 was also tested in a promonocytic cell line, as well as in latently infected microglial cells [51]. The relevance of this work stands in the fact that microglial cells represent the main cell type harboring HIV-1 in the brain [52]. Furthermore, persistently infected astrocytes were also adopted as experimental model [53]. 

Overall, literature data indicate that the CRISPR-Cas system offers several advantages in targeting latent proviral DNA. However, issues remain unsolved when trying to apply this strategy in vivo. In particular, as a true marker of HIV latency is lacking, it is difficult to specifically target latent infected cells [54]. Furthermore, infected cells may be hidden in anatomical reservoirs, which are difficult to reach by the commonly used delivery systems. Finally, it is still under debate whether eradication of HIV from all the infected cells is really needed or if it is sufficient to clear a percentage of them to achieve a functional cure [18]. 

In 2016, the first in vivo proof of concept was carried out in mice and rats by showing that targeting the provirus is feasible in different cells and tissues [55]. The authors of this study injected HIV-1 Tg26 mice with two successive tail-vein injections of AAV9 vectors encoding for SaCas9 together with LTR- and *gag*-targeting gRNAs, then analyzed via PCR the DNA extracted from different tissues. They demonstrated that in all the analyzed tissues the region encompassing the targeted HIV sequence presented a deletion. The results were confirmed in a rat model, showing excision of the targeted sequence and up to 80–90% reduction of *gag* and *env* RNA, respectively, in circulating lymphocytes.

In a following study, the same research group investigated in vivo the efficiency of all-in-one AAV-SaCas9 vectors with a quadruplex of gRNAs, to obtain an increased suppression of viral transcription and replication by targeting LTRs and structural/functional genes, as presented in Section 2.4 [37]. A single injection of the vectors in Tg26 mice resulted in deletions of the targeted sequence in the liver, bone marrow, and spleen, with an increased cleaving efficiency extended to other tissues after a second injection, and no apparent off-target effects. As the Tg26 model does not entirely recapitulate HIV infection/latency, the authors took advantage of an NCr nude mouse, infected with an HIV-enhanced luciferase reporter (HIV-eLuc) and with the AAV-SaCas9 vector. Then, they adopted the more clinically relevant BLT mouse model. These mice were infected with the HIV-eLuc reporter virus via intravaginal and intraperitoneal route. After the delivery of AAV-SaCas9 vector by intravenous or intravaginal route, the presence of the proviral DNA in different tissues was analyzed, demonstrating the efficiency of the excision [37]. Points of strength of this work are the use of different mouse models and of a gRNA quadruplexing approach, which gave encouraging pre-clinical results. However, before moving to clinical applications, more clinically relevant models must be adopted. 

Under this respect, Mancuso and co-workers explored in vivo the antiviral activity of an ad hoc developed AAV9-saCas9 platform by employing non-human primates (rhesus macaques) challenged with the simian immunodeficiency virus (SIV) [32]. The authors showed a convincing reduction in the percentage of SIV intact DNA in the blood, with excision efficiencies ranging from 37% to 92% in different animals, as well as in different tissues, including known tissue reservoirs, such as lymph nodes, spleen, bone marrow, and brain.

**Figure 4:**
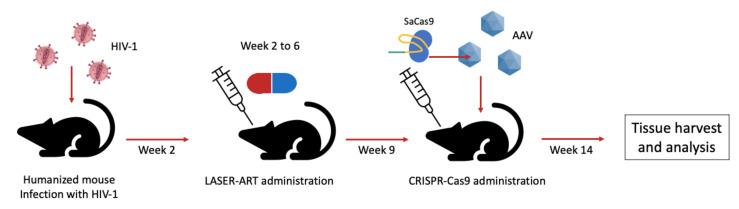
Representation of the experimental setup used in Dash PK et al. [31]. AAV stands for Adeno-Associated Virus based vectors.

A fascinating study from Dash and co-workers [31] demonstrated in an in vivo proof of principle how the combination of an innovative and highly penetrating ART, followed by the administration of the CRISPR-Cas9 system, could lead to the elimination of HIV in a mouse model. In this report, the authors first established a humanized mouse model infected by HIV. Mice were then treated with a novel pharmaceutical strategy, named long-acting slow-effective release (LASER) ART. LASER ART is characterized by an enhanced lipophilicity to penetrate viral reservoirs, along with a slow release, allowing a lower frequency of administration. This approach, however, was not sufficient to eliminate the virus from the latently infected cells, so three weeks after the last drug treatment, the mice received an intravenous delivery of an AAV-9 vector encoding for the SaCas9 along with two gRNAs targeting the LTR and *gag* region, respectively. Five weeks after the AAV administration, plasma viral load was evaluated (Figure 4). 

While, in all the control mice (treated with the LASER-ART alone) viral rebound was observed, in two out of the seven mice treated with both the LASER-ART and AAV9-CRISPR-Cas9, viral load was undetectable. The analysis of viral DNA and RNA in different tissues showed a more efficient DNA copy number reduction in dual treated mice compared with the mice treated with LASER-ART alone or with AAV9-CRISPR-Cas9 alone. Importantly, neither viral DNA nor viral RNA were found in two mice in which the viral rebound did not occur. The experiment was replicated in a second and third separate set of animals with a total of seven out of sixteen mice, which showed no viral rebound [31]. The study has certain limitations. First, HIV was eliminated only in a fraction of animals. Second, the relatively short follow-up after CRISPR administration could have limited the observation of viral rebounds occurring at later time points. Finally, the extent of the viral reservoir established in this experimental model might not perfectly recapitulate what occurs in humans. Nevertheless, this work convincingly shows that combining highly effective antiretroviral therapies with the novel gene editing approaches could be a promising and effective strategy for tackling the proviral HIV.

## 4. The Catalytically Inactive Cas9 as a Modulator of Provirus Transcription

The CRISPR system, besides the direct editing of HIV viral sequences, may be useful in the context of latent infection to modulate the provirus transcription. In fact, latently infected cells are not recognized and eliminated by the immune system and their stochastic reactivation may lead to the reactivation of viral replication and rebound. Two strategies are currently explored to solve this issue. The first one is known as the “shock and kill” approach and is based on the reactivation of the latent virus by specific treatments, so that the immune system can recognize/eliminate the infected cells [56]. Alternatively, viral replication itself would kill them. The second method is the so-called “block and lock” approach, which is aimed at permanently blocking virus reactivation [57].

Limitations of these strategies, which are not yet a therapeutic reality, but were investigated in different studies, are represented by the difficulties to reach all the latently infected cells. Furthermore, in the case of the “shock and kill” approach, the “killing” part is still not efficient enough [56]. Importantly, both these approaches entail the administration of molecules, such as latency reversal agents (LRAs) and Tat inhibitors, which pose a risk of toxic off-target effects. Interestingly, drugs have been replaced by the catalytically inactive form of the Cas9 [58]. For example, a dCas9 fused with a Krüppel-associated box (KRAB) transcriptional repression domain has been adopted to inhibit proviral reactivation. By using stably expressing dCas9-KRAB lymphoblastoid T cells, it has been demonstrated that, upon cell stimulation by LRAs, HIV-1 expression was reduced up to 60% with respect to the control after the delivery of specific gRNAs designed to direct the dCas9 to the LTR promoter regions. This effect was related to the presence of repressive epigenetic modifications, suggesting the possibility of engineering the CRISPR system for a “block and lock” approach [11].

Regarding the application for a “shock and kill approach”, one of the first studies investigating this possibility is the one from Zhang and co-workers [59]. Those authors demonstrated that the use of an LTR-targeting dCas9, along with a synergistic activator mediator (SAM) system, were able to increase the activity of the LTR-driven gene expression in the human epithelial cell line TZM-bI (HeLa-derived cell line containing beta-galactosidase and luciferase reporter genes under the transcriptional control of the LTR) and in the human embryonic kidney cells HEK-293T. The results were confirmed in several cell models of HIV-1 latency as well as in a microglial cell line. Following studies further investigated the potency of this approach, demonstrating for example: (i) its ability to stimulate the production and release of infectious viral particles in latent cell models [60]; (ii) the possibility to use a combination of the dCas9-SAM along with histone deacetylase inhibitors or other latency-reversal compounds to synergistically enhance the provirus activation in cell models of HIV latency [61]; (iii) a different efficiency in relation to which LTR sequence was targeted, identifying in particular a NF-κB binding site-targeting gRNA as one of the most potent in inducing the provirus activation [62]. A more recent study from Zhang and co-workers, extensively analyzed possible off-target effects resulting in altered transcriptional profiles related to the use of the dCas9-SAM [63]. The authors demonstrated that only two genes were significantly upregulated out of the tested ones. Even though this investigation was limited by the small replicate size, and by the fact that further tests in other cell models (and possibly in an in vivo setting) would be needed, it was the first evidence indicating the safety of this strategy for a possible future therapeutic application.

Overall, the use of dCas9 over the pharmacological approach has the advantage that, being sequence-specific, it could limit off-target toxicity effects. Moreover, the dCas9-mediated “shock and kill” provirus activation seems to be more efficient than the one achieved by drug treatment. On the other hand, the main disadvantage is that the technique is still relatively new and up to now it has been tested only in vitro, so there is still few information on its possible clinical relevance [12].

## 5. CRISPR Screening to Find Host Dependency Factors Involved in HIV-1 Latency

In the previous sections, we presented some possible therapeutic strategies of the CRISPR system by targeting the latent provirus. However, this technology may be employed in the context of HIV latency for other applications [64]. One of the most interesting and recent ones is its use to screen for new host dependency factors (HDFs), i.e., specific factors of the host infected cells which are necessary for the virus to establish and maintain the infection. In particular, the identification of new HDFs in HIV-1 infected cells is important both to understand better the mechanisms of HIV infection and persistence in the cells as well as to discover new possible antiviral therapy target [65].

**Figure 5:**
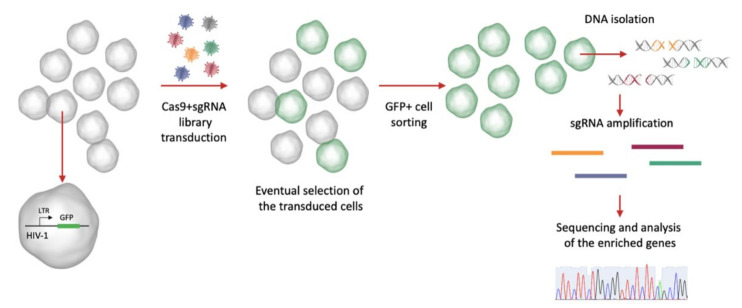
A possible CRISPR screen strategy applied to a latency model. The latently infected cell model may harbor a fluorescent reporter (e.g., the green fluorescent protein, GFP) under the transcriptional control of the viral long terminal repeat (LTR). The cells are transduced with the Cas9 and the sgRNA library, and when a gene involved in the maintenance of the latent provirus is targeted, the provirus activates; thus, the cells start to express the reporter. The GFP-positive cells are sorted (positive selection), the genomic DNA is extracted, and the sgRNA regions are amplified. At this point, the relative sgRNA abundance is compared with a control population, to identify the enriched genes.

The CRISPR knockout screening system, in principle, consists in the generation of a library of sgRNAs designed to target different genes with redundancy (meaning that more sgRNAs may target the same gene), which is delivered to the cells along with the Cas9 in a pooled manner. The targeted cells are then selected, respectively, by a positive selection, if the genetic perturbation allows the cells to survive or proliferate under a selective pressure, or a negative selection, if the perturbation causes the cells to be depleted over time [66]. By extracting the DNA from the cells and sequencing the selected regions, it is possible to understand which targeted genes resulted in the observed phenotype under the selected conditions (Figure 5).

The CRISPR screens have the advantage over other methods, such as the shRNA screens, to be more robust and specific, with a lower false discovery rate [67]. 

When using the keywords “CRISPR screen HIV” in the NCBI database PubMed, roughly 60 publications can be found. Selecting among these, the ones strictly based on the use of CRISPR screens to find new HIV-1 HDFs, are 15 papers, 1 review, and 2 commentaries, reported since 2016–2017, when the first studies appeared [29,68]. Out of these, six studies specifically focused on the use of this technology to find new cellular factors to promote and maintain viral latency (Table 1).

The first to apply a genome-wide CRISPR screen method for this purpose were Jin and co-workers [69], who took advantage of the previously developed GeCKO lentiCRISPRv2.0 pooled sgRNA library [70] to transduce the Jurkat-T-cell-derived C11 cell model [76], harboring a latent HIV-1 provirus containing the GFP reporter. After the delivery of the CRISPR screen vectors, they analyzed those genes that were enriched in the four-time sorted GFP-positive cells, identifying three top-scoring genes, *SUV39H1*, *TSC1*, and *DEPDC5*. SUV39H1, a chromatin modulator, was known from previous studies as a latency-promoting factor. By contrast, TSC1 and DEPDC5 potential functions in HIV latency were analyzed in this study again in C11 cells. The authors identified these proteins as suppressors of the cellular mTORC1 protein complex [69]. mTORC1 is known to regulate different cellular processes such as growth and metabolism, which can be modulated by viral infection [77]. Thus, its suppression in HIV latency makes sense. Although interesting in its conclusions, one of the main limitations of this investigation is the fact that cell lines, as the one adopted, might be different in their array of metabolism modulators and/or in their involvement in HIV latency from primary cells [78].

Another screening strategy was employed by Li and collaborators, which stably transduced a 2D10 Jurkat-derived HIV-1 latency reporter cell line [79] with the dCas9-KRAB transcriptional suppressor (see Section 4 of this review) under the control of a doxycycline-inducible promoter (called CRISPRi, that stands for CRISPR interference, reporter cell line) [71]. Then, the authors stably transduced these cells with a whole genome sgRNA library, administered doxycycline to allow the expression of the dCas9 complex, and then sorted cells, which resulted GFP-positive. To increase the sensitivity of the system, the screen was repeated four times, each time enriching the sgRNA library with the sequences identified from the previous round, in a procedure that was defined Reiterative Enrichment and Authentication of CRISPRi Targets (REACT). This approach allowed the identification of six significantly enriched genes: *NFKBIA*, *CYLD*, *GON4L*, *PSMD1*, *PSMD3*, and *PSMD8*. The first two genes were known to encode for HIV-1 transcription suppressors, thus their effect on the provirus reactivation was expected, while the last four were novel and previously unreported, encoding respectively for a transcriptional co-repressor (GON4L) or for proteasome subunits (PSMD1, PSMD3, and PSMD8). The authors provide evidence for a link between the observed provirus reactivation and the block of proteasomal degradation of ELL2, a factor involved in Tat-mediated transactivation [80]. Overall, the advantage of this system is related on the possibility to enrich the signal over the background thanks to its iterative nature. However, it should be kept in mind that this approach could result, at the same time, in an under-representation of certain genotypes.

Other studies adopted the Cas9 knockout screens by using a sub-pool of sgRNAs instead than a genome-wide one. For example, Huang and co-workers [72], with the aim of focusing on possible nuclear cell factors modulating provirus latency, adopted a sub-pool library, targeting nuclear proteins applied to the J-Lat A2 latency cell model. The authors were able to identify the MINA53 histone demethylase as a latency-promoting factor.

Rathore and collaborators [73] exploited the same GeCKO sgRNA library and a similar protocol as Jin and co-workers [69], by transducing the J-Lat 10.6 T lymphoblastoid cells, and confirmed the results in a second latency cell model. They identified the following genes: *IWS1*, *POLE3*, *POLR1B*, *PSMD1*, and *TGM2*. IWS1 was characterized in previous studies as a transcriptional repressor. On the other hand, PSMD1 was already reported by Li and colleagues [71], who hypothesized the disruption of the proteasome subunits as mechanism by which PSMD1 depletion could reverse latency. POLE3, POLR1B, and TGM2 were novel identified factors. POLE3 has a heterochromatin-remodeling role favoring histone deposition and chromatinization; POLR1B is a component of RNA Pol I, and its silencing could lead to the activation of p53 and a generalized gene reactivation including that of the provirus. However, pharmacological inhibition of POLR1B did not lead to HIV-1 reactivation, thus requiring further investigations. Finally, TGM2 is a cross-linking enzyme, which is involved in transcriptional repression. Interestingly, the interpretation of the functions and pathways in which the identified genes are involved along with the observation that PSMD1 leads to the activation of the deubiquitinating enzyme UCH37, led the authors to hypothesize that deubiquitination might play a role in latency reversal. They performed a second CRISPR screen by specifically targeting deubiquitinase genes and identified three new deubiquitinases involved in HIV-1 latency, thus showing the importance to integrate the information coming from the screens with further assays to understand how the identified factors are interconnected and contribute to maintain the provirus latent state [73].

Krasnopolsky and co-workers [74] used the sgRNA GeCKO library, with a slightly different approach than the other studies. Those authors adopted Cas9 stably expressing Jurkat cells transduced with blue fluorescent protein (BFP)-expressing lentiviruses and then with the sgRNA library. Cells were allowed to return to a resting state, as determined by the decrease of BFP expression, and sorted. The comparison between BFP-depleted sorted cells and the unsorted control led to the identification of the highest-ranked enriched gene, *ZNF304*, coding for a KRAB-containing zinc finger protein which is able to recruit a repressive complex to the HIV-1 promoter, silencing it. 

The most recent one of the publications reported in Table 1 [75], took advantage of the same cell model and sgRNA library used by Jin and co-workers [69], and demonstrated a novel possible role for *PEBP1*. This gene encodes for a kinase inhibitor protein (called Raf1 kinase inhibitor protein, or RKIP) involved in the MAPK and NF-κB signaling pathways. It is known that NF-κB inactivation favors latency. However, it is not clear how this happens during the latency establishment. The authors propose that PEBP1 could induce latency by acting upstream of the NF-κB pathway by preventing the translocation of this transcriptional factor from the cytoplasm to the nucleus. From this screening, the *FKBP3* gene was also identified. The FKBP protein was found to bind HIV-1 LTR, promoting histone deacetylation and, thus, latency [81].

Altogether, data in the literature provide important insights on the cellular factors or pathways that might play a role in HIV-1 latent infection. However, some important issues need to be taken into consideration. First, these studies were performed in cell lines in which the mechanisms of latency may differ from what happens in cART-suppressed individuals. Moreover, most of the adopted cell lines are clonal, thus sharing the same provirus sequence and integration sites in clear contrast to the in vivo conditions, where the integration sites, proviral copy number, and sequences may be heterogeneous [82]. Interestingly, recently developed techniques, such as barcoded HIV viruses [83,84], by allowing the tracking of viral integration site and reactivation, showed that the use of different LRAs may lead to a stronger or weaker provirus reactivation, depending on the integration site. Thus, the combination of HIV barcoding, and of other approaches, such as single-cell RNA sequencing [85], with the CRISPR screens could overcome these limitations and provide further insights on the molecular interplays involved in viral latency maintenance and reactivation.

## 6. Concluding Remarks

The CRISPR-Cas system has revolutionized many technological applications in biology in the few years since its discovery, including in the HIV-1 research field. Gene editing technologies, in particular, seem to provide an answer to the question posed by the persistence of HIV-1 infection in the host cells, by allowing the specific and direct targeting of the provirus or of host-genes involved in viral replication.

In this review, we presented some of the most relevant studies that adopted the CRISPR-Cas system to tackle the latent proviral genome and to disrupt/eliminate it from the cells in vitro as well as in animal models. In some cases, a reduction higher than 90% in the viral copy number in patient-derived cells [49] and in non-human primates [32] was accomplished. It was also demonstrated that the system can efficiently target transcriptionally silent genomic regions and both the integrated and the pre-integrated viral DNA [47], being effective in latently as well as in de novo infected cells [48].

Paramount to the success of the approach is the careful design of the sgRNA targets, and the majority of the studies have highlighted the need of selecting highly conserved viral sequences as well the importance of using a multiplexing approach. The latest allows, on the one hand, to develop a broad-spectrum acting system to cover the inter- and intra-patient variability of the provirus [42]. On the other hand, it should limit/avoid viral escape [41]. Importantly, several nucleases and different delivery systems, such as AAV [37] and lentiviral vectors [36], can be adopted to effectively reach the cells/tissue of interest. Moreover, the combination of an effective delivery system along with novel and highly penetrating antiretroviral therapies has been shown to clear the virus, at least in a subset of animals, from different tissues in an in vivo mouse model [31]. This finding supports the hypothesis that it is possible to achieve viral clearance by combining different efficient therapeutic strategies, even though further improvements are needed.

The potential and versatility of the approach is further supported by the diversity of actions employed up to now, which are not limited to the direct targeting of the provirus and its disruption or excision, but also to the possibility of using the catalytically inactive Cas9 to modulate, at a transcriptional level, the latent provirus in the “shock and kill” or “block and lock” approaches [12]. Importantly, these dCas9-based systems seem to display higher potency in activating the proviral DNA as well as more targeted activity and, thus, lower toxic off-target effects than the canonical LRAs.

Despite the promising results obtained so far, the fact that the mechanisms of latency are still not fully understood makes developing new approaches more challenging. The CRISPR-Cas system has also found useful application in this context. Indeed, CRISPR screens in genome-wide knockout experiments [68] allow the identification of novel host-dependent factors implicated in the establishment and maintenance of the latent provirus. In this review, we have presented recent studies, which have employed different HIV-1 latency cell models and CRISPR screening strategies, to identify the involvement of previously not identified host factors in viral latency. By taking these studies together, it is clear that the virus and the host cell interact and modulate each other and that different cellular pathways are involved in the provirus latency. Of course, it should be kept in mind that the adoption of different cell lines, where the latent phenotype was achieved by different strategies, might have contributed to the identification of different factors [82].

Overall, the CRISPR-Cas system has shown promising results towards future applications in a clinical setting, possibly in combination with other therapies. However, before thinking about the clinical translation of this tool, one of the greatest limitations that must be addressed remains the delivery of the CRISPR system to the latently infected cells, which lack a clear marker and are difficult to access in vivo. Moreover, it is necessary to find appropriate models that can recapitulate what happens in a heterogeneous context, such as the viral reservoir in cART-treated patients.

## Figures and Tables

**Table 1:** Selected reports, which used CRISPR screens to discover new host cell factors involved in HIV-1 latency.

References	Strategy Employed	Cell Line	Gene(s) Identified	Gene Function
Jin S, et al. [69]	Genome-scale CRISPR Knock-Out (GeCKO) lentiCRISPRv2.0 genome-wide pooled sgRNA library [70]	Jurkat-derived C11 cell line	*SUV39H1*, *TSC1* and *DEPDC5*	Heterochromatin modulation, mTOR signaling pathway modulator
Li Z, et al. [71]	Tet-On dCas9-KRAB-mCherry stable cells transduced with a genome-wide pooled sgRNA library, application of Reiterative Enrichment and Authentication of CRISPRi Targets (REACT)	Jurkat-derived 2D10 cell line	*PSMD1*, *NFKBIA*, *CYLD*, *GON4L*, *PSMD3*, and *PSMD8*	Transcriptional suppression/co-repression, proteasome subunit
Huang H, et al. [72]	Lentiviral transduction of a sgRNA sub-pool library targeting nuclear proteins	J-Lat A2 (Tat-GFP) cell line	*MINA53*	Histone demethylase
Rathore A, et al. [73]	Genome-scale CRISPR Knock-Out (GeCKO) lentiCRISPRv2.0 genome-wide pooled sgRNA library [70]	J-Lat 10.6 cell line	*IWS1*, *POLE3*, *POLR1B*, *PSMD1*, and *TGM2*	Transcriptional repressor, proteasome subunit, heterochromatin remodeling, component of RNA Pol I, enzyme which cross-links proteins
Krasnopolsky S., et al. [74]	Genome-scale CRISPR Knock-Out (GeCKO) lentiCRISPRv2.0 genome-wide pooled sgRNA library [70]	Jurkat T cell line (transduced with a HIV-BFP vector)	*ZNF304*	KRAB-containing zinc finger protein
Yang X, et al. [75]	Genome-scale CRISPR Knock-Out (GeCKO) lentiCRISPRv2.0 genome-wide pooled sgRNA library [70]	Jurkat-derived C11 cell line	*PEBP1*	Kinase inhibitor protein involved in MAPK and NF-κB signaling pathways
